# Reference gene selection for qRT-PCR analyses of luffa (*Luffa cylindrica*) plants under abiotic stress conditions

**DOI:** 10.1038/s41598-021-81524-w

**Published:** 2021-02-04

**Authors:** Min-dong Chen, Bin Wang, Yong-ping Li, Mei-juan Zeng, Jian-ting Liu, Xin-ru Ye, Hai-sheng Zhu, Qing-fang Wen

**Affiliations:** Fujian Key Laboratory of Vegetable Genetics and Breeding, Crops Research Institute, Fujian Academy of Agricultural Sciences, Vegetable Research Center, Fujian Engineering Research Center for Vegetables, Fuzhou, 350013 Fujian China

**Keywords:** Molecular biology, Plant sciences

## Abstract

Selecting suitable internal reference genes is an important prerequisite for the application of quantitative real-time PCR (qRT-PCR). However, no systematic studies have been conducted on reference genes in luffa. In this study, seven reference genes were selected, and their expression levels in luffa plants exposed to various simulated abiotic stresses [i.e., cold, drought, heat, salt, H_2_O_2_, and abscisic acid (ABA) treatments] were analyzed by qRT-PCR. The stability of the reference gene expression levels was validated using the geNorm, NormFinder, BestKeeper, and RefFinder algorithms. The results indicated that *EF-1α* was the most stably expressed and suitable reference gene overall and for the heat, cold, and ABA treatments. Additionally, *UBQ* expression was stable following the salt treatment, whereas *TUB* was identified as a suitable reference gene for H_2_O_2_ and drought treatments. The reliability of the selected reference genes was verified by analyzing the expression of copper/zinc superoxide dismutase (*Cu/Zn-SOD*) gene in luffa. When the most unstable reference genes were used for data normalizations, the resulting expression patterns had obvious biases when compared with the expression patterns for the most ideal reference genes used alone or combined. These results will be conducive to more accurate quantification of gene expression levels in luffa.

## Introduction

Quantitative real-time PCR (qRT-PCR), which is a molecular technique enabling researchers to quantitatively analyze nucleic acids, was developed based on the common PCR technique and has been widely used in many research fields (e.g., agriculture, medicine, microbiology, and molecular diagnostics)^1–3^. The advantages of qRT-PCR include its specificity, sensitivity, and reproducibility as well as the fact it can be completed very quickly and efficiently, making it appropriate for assessing gene expression and rapidly quantifying mRNA transcripts^4,5^. However, the accuracy of this quantitative analysis can be influenced by the initial template quantity, the RNA quality, and the enzymatic reaction efficiency^6,7^. Therefore, normalizing target gene expression levels against the data for suitable reference genes is essential for minimizing the biases associated with qRT-PCR results.

Reference genes, also known as housekeeping genes, refer to genes that are generally stably expressed in different tissues and developmental stages as well as under various experimental conditions. Common reference genes include 18S ribosomal RNA (*18S*), β-tubulin (*TUB*), α-tubulin (*TUA*), elongation factor 1 alpha (*EF-1α*), ubiquitin (*UBQ*), glyceraldehyde-3-phosphate dehydrogenase (*GAPDH*), and actin (*ACT*) genes^8,9^. However, many studies have confirmed there is a lack of universal reference genes for plants. The suitability of specific reference genes depends on the experimental conditions, and the use of unstably expressed reference genes will likely produce biased results and false-positives^10,11^. Gutierrez et al.^12^ revealed that several genes commonly used as reference controls in *Arabidopsis thaliana* studies are unstably expressed. Additionally, using non-validated reference genes can lead to 100-fold changes in target gene expression levels. Therefore, selecting and validating reference genes under diverse experimental conditions is crucial before a meaningful qRT-PCR analysis can be performed. There are currently several publicly available online statistical tools, including geNorm, NormFinder, BestKeeper, and RefFinder, developed to identify a series of appropriate reference genes as internal controls for normalizing qRT-PCR data.

*Luffa cylindrica* (i.e., luffa), which belongs to the family Cucurbitaceae, is one of the most important vegetables and widely used medicinal plants in China. Many luffa plant parts and components (e.g., mature fruit, leaf, stem, root, seed, flavonoid and polysaccharide) have been used in cough remedies and for treating rhinitis and lumbago. Therefore, there may be a considerable market for luffa products, making the commercial production of luffa an economically important industry^13^. However, the early luffa fruit development stage generally coincides with a period of heavy rainfall and high temperatures, which can damage seedlings (e.g., rotting roots and leaves), leading to considerable decreases in productivity. Plants are exposed to a variety of abiotic and biotic stresses that can restrict growth and development, with abiotic stresses proven to adversely affect crop yield and quality^14^. Consequently, the mechanism regulating the adaptation of luffa to abiotic stresses must be elucidated, including the related genes, which necessitates the selection of suitable reference genes for gene expression analyses. To date, the research regarding reference genes appropriate for luffa has been quite limited (i.e., only *18S*^15^ ), and the stability of reference genes has rarely been reported. In the present study, five common reference genes (*ACT*, *TUA*, *TUB*, *EF-1α*, and *GAPDH*) were cloned from luffa seedlings. These genes along with two previously cloned reference genes, *UBQ*^16^ and *18S*, were analyzed to identify the most reliable reference genes for normalizing target gene expression via qRT-PCR. Experimental samples were exposed to various stresses, including heat, cold, H_2_O_2_, salt, abscisic acid (ABA), and drought. Four algorithms (geNorm, NormFinder, BestKeeper, and RefFinder) were applied to evaluate the reference genes and to identify the most stably expressed genes under diverse conditions. Furthermore, to assess the utility of the validated reference genes, the expression levels of copper/zinc superoxide dismutase (*Cu/Zn-SOD*) gene under the above conditions were examined. The results of these analyses may provide the basis for future qRT-PCR-based investigations of the transcription of important functional genes in luffa.

## Results

### Isolation of reference genes

**Figure 1:**
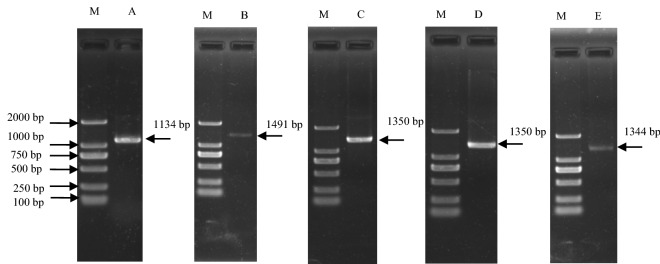
Agarose gel electrophoresis analysis of the ORFs for five reference genes in luffa. M: Marker 2000; A: *ACT*; B: *GAPDH*; C: *TUA*; D: *TUB*; E: *EF-1α.* The gels were cropped and the full-length gels were presented in Supplementary Fig. 1, 2.

The sequencing results indicated that the full-length ORF sequences of the *ACT*, *TUA*, *TUB*, *EF-1α*, and *GAPDH* genes were 1134, 1350, 1350, 1344, and 1491 bp long, respectively (Fig. 1). A BLAST analysis revealed that the ACT amino acid sequence was more than 98% identical to *Cucumis melo* (XP_008447829.1), *Momordica charantia* (XP_022143797.1), and *Theobroma cacao* (XP_017977121.1) sequences. The TUA amino acid sequence was more than 98% identical to *Momordica charantia* (XP_022153822.1), *Cucurbita moschata* (XP_022944288.1), and *Vitis vinifera* (XP_002285721.1) sequences. The TUB amino acid sequence was more than 96% identical to *Cucumis sativus* (XP_004139067.1), *Momordica charantia* (XP_022153267.1), and *Brassica oleracea* (VDD33972.1) sequences. The EF-1α amino acid sequence was more than 96% identical to *Cucurbita moschata* (XP_022959419.1), *Gossypium hirsutum* (XP_012468306.1), and *Vitis vinifera* (XP_002284924.1) sequences. The GAPDH amino acid sequence was more than 96% identical to *Cucurbita moschata* (XP_022937833.1), *Cucumis melo* (XP_008456541.1), and *Momordica charantia* (XP_022143215.1) sequences. These sequence matches confirmed that the obtained sequences corresponded to the *ACT*, *TUA*, *TUB*, *EF-1α*, and *GAPDH* genes.

### Primer specificity and amplification efficiency analysis

**Figure 2:**
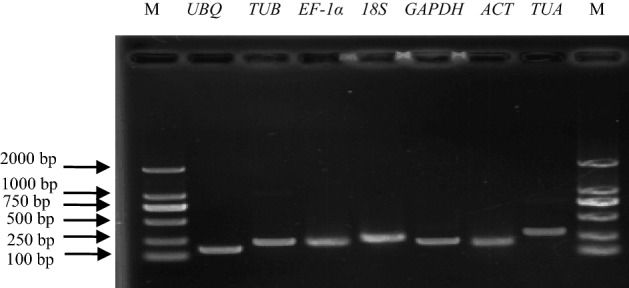
Agarose gel electrophoresis analysis of primer specificity.

**Figure 3:**
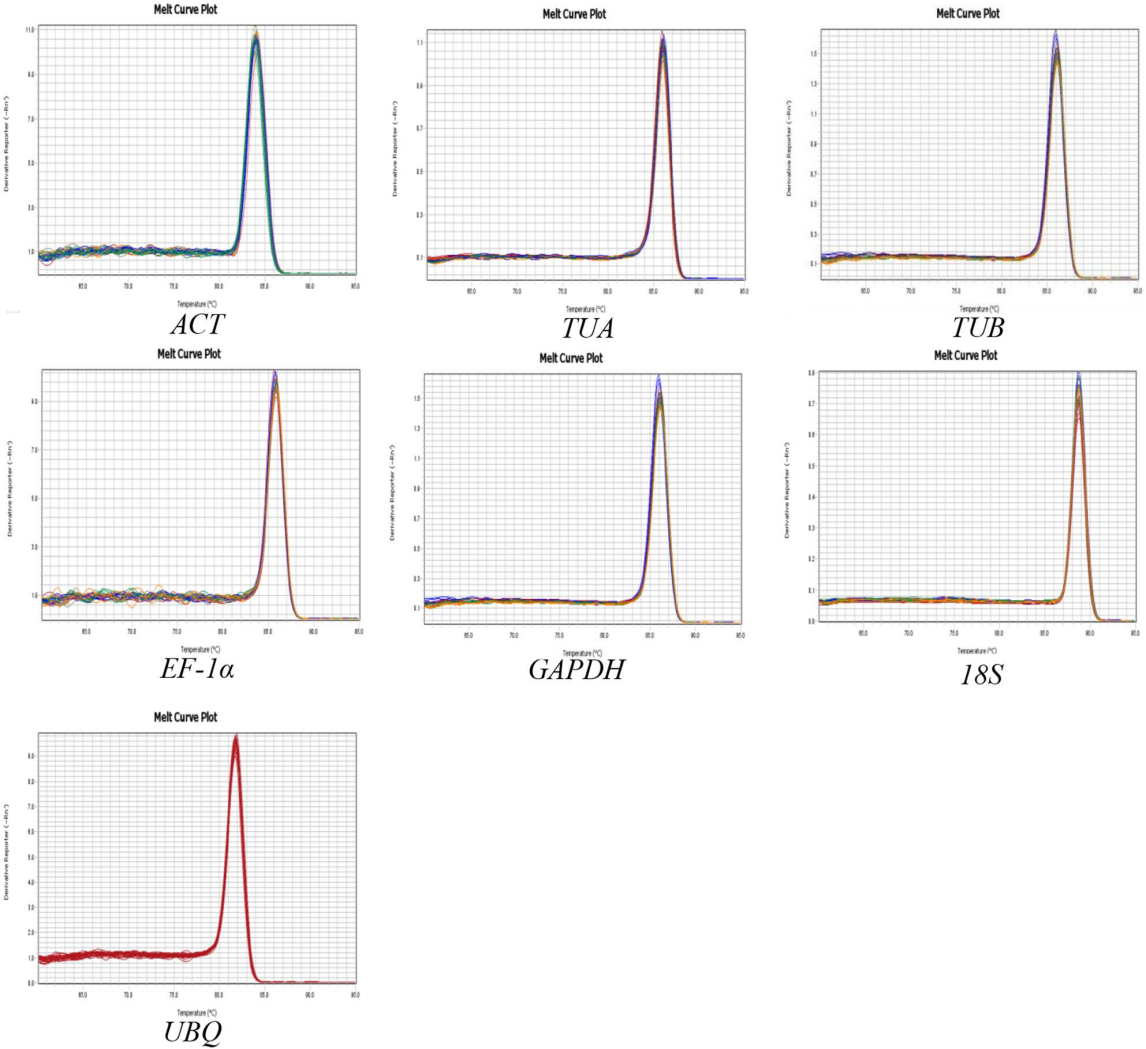
Melting curve analysis of seven reference genes.

Seven genes (*ACT*, *TUA*, *TUB*, *EF-1α*, *GAPDH*, *UBQ*, and *18S*) were selected as candidate reference genes. Information regarding these genes and their qRT-PCR primer pairs are summarized in Table 1. Gel electrophoresis and melting curve analyses were used to determine primer specificity. All primers amplified a single amplicon of the expected size (Fig. 2). The seven candidate reference genes under various abiotic stress conditions produced a single peak during the melting curve analysis (Fig. 3). These results indicated the primers for these genes were highly specific. The amplification efficiencies for the seven candidate reference genes ranged from 97.6% (*TUB*) to 104.2% (*18S*), and the correlation coefficients varied from 0.988 (*ACT*) to 0.997 (*18S*) (Table 1). Therefore, all primers were appropriate for qRT-PCR analyses.

**Table 1:** Details on primers used for qRT-PCR analysis.

Gene	Gene description	Genbank ID	Primer sequences (forward primer/reverse primer, 5′–3′)	Product length (bp)	Amplification efficiency (%)	Correlation coefficient R^2^
*UBQ*	Polyubiquitin	KR349345	5′-TGCTTCGTCTCAGGGGTGG-3′ 5′-GTCCTGAATTTTAGCTTTCAC-3'	116	102.5	0.991
*TUB*	Beta-tubulin	MN548043	5′-GTGCTGGTAATAACTGGG-3' 5′-GGGAAGACGGAGAAAGTA-3'	226	97.6	0.992
*EF-1α*	Elongation factor-1α	MN548044	5′-TCAAGAAGGTCGGATACA-3' 5′-ACAGGGACAGTTCCAATAC-3'	223	97.8	0.991
*18S*	18S ribosomal RNA	KM656452	5′-CTGGTCTTTTCGGATGAT-3' 5′-CCTTTACGCCCAGTCATT-3'	266	104.2	0.997
*GAPDH*	Glyceraldehyde-3-phosphate dehydrogenase	MK766385	5′-TTATCAACCCCACTACCA-3' 5′-TTCCTTCACCAAACACTC-3'	203	98.6	0.995
*ACT*	Actin	MN548045	5′-GTCGCCCTCGCCATACAG-3' 5′-CTCTTCGGGAGCAACACG-3'	194	101.8	0.988
*TUA*	Alpha-tubulin	MK778379	5′-ATTGGACAGGCTGGGATT-3' 5′-TTGGCAGCATCTTCTTTT-3'	278	100.5	0.993
*LcCu/Zn-SOD1*	Copper/zinc superoxide dismutase	KP178922	5′-CACAGGAAAGATGGTGAAGG-3′ 5′-CCAGCAGGGTTGAAATGT-3’	210

### Expression profiles of reference genes

**Figure 4:**
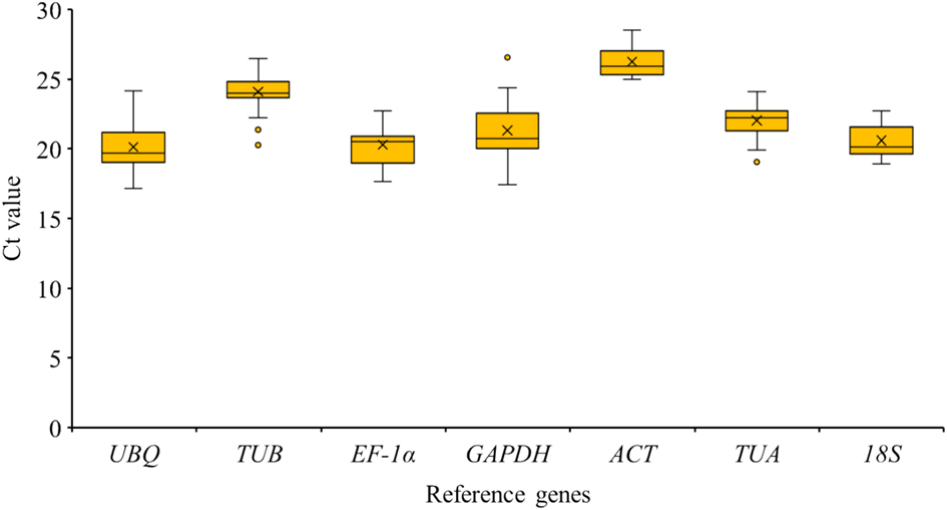
Distribution of Ct values among the seven candidate reference genes across all analyzed luffa samples. The box indicates the 25th and 75th percentiles, with the line across the box representing the median. The whiskers and asterisks represent the 95% confidence intervals and outliers, respectively.

The expression levels of the seven reference genes were calculated based on the Ct values (Supplementary Table S1), with lower Ct values corresponding to higher expression levels. Box plots were used to present the distribution of the Ct values (Fig. 4). The average Ct values across all samples varied from 20.13 to 26.25, reflecting the diversity in expression levels. Five genes (*UBQ*, *EF-1α*, *18S*, *TUA*, and *GAPDH*) were highly expressed, with average Ct values between 20 and 22, whereas two genes (*TUB* and *ACT*) were moderately expressed, with Ct values between 24 and 26. Of all analyzed genes, the highest and lowest expression levels were calculated for *UBQ* (mean Ct of 20.13) and *ACT* (mean Ct of 26.25), respectively. Additionally, the most and least variable expression were detected for *GAPDH* and *TUA*, respectively, suggesting that *GAPDH* was the most unstably expressed gene, whereas *TUA* was the most stably expressed gene.

### Expression stability of reference genes

In this study, gene expression was examined in plants exposed to various abiotic stress conditions (i.e., heat, cold, H_2_O_2_, salt, ABA, and drought stresses). Four algorithms (geNorm, NormFinder, BestKeeper, and RefFinder) were used to analyze the expression of relatively stable reference genes.

### geNorm analysis

**Figure 5:**
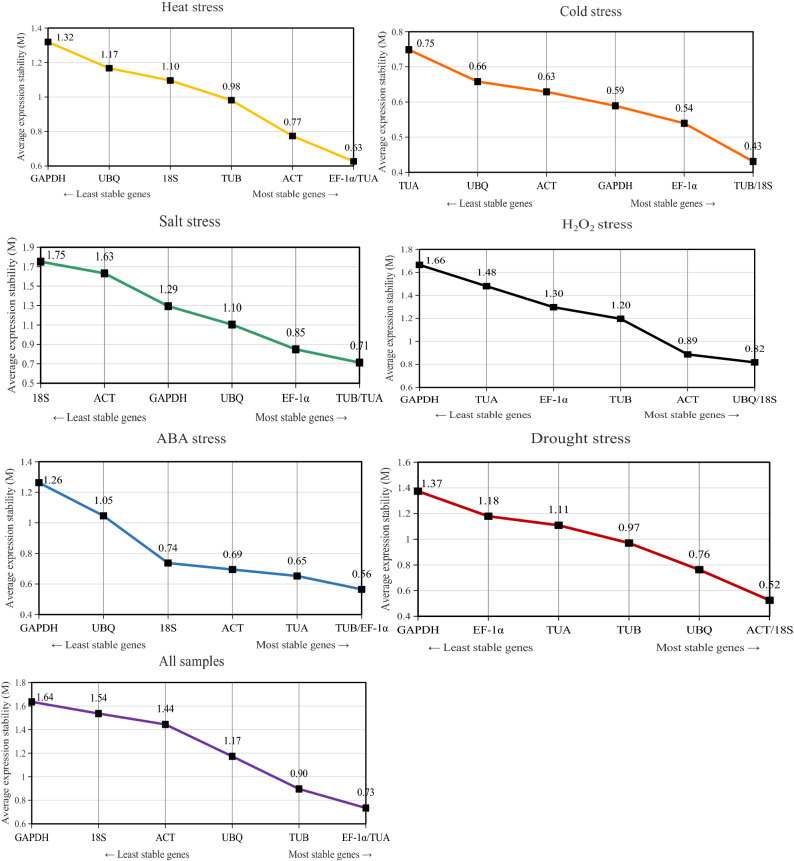
Expression stability of seven reference genes in luffa under different conditions based on a geNorm analysis.

The geNorm algorithm evaluates the stability of reference gene expression by calculating the average M value, with values less than 1.5 indicating stable expression. Thus, the gene with the lowest M value is considered to be the most stably expressed. In this study, the most stably expressed reference gene differed among the stress conditions. For the heat treatment, *EF-1α* and *TUA* were the most stably expressed genes, with an M value of 0.63, whereas *GAPDH* was the least stably expressed gene, with an M value of 1.32. For the cold treatment, *TUB* and *18S* (M = 0.43) were the most stably expressed genes, whereas *TUA* (M = 0.75) was the least stably expressed gene. Regarding the H_2_O_2_ treatment, the most stably expressed genes were *UBQ* and *18S* (M = 0.82) and the least stably expressed gene was *GAPDH* (M = 1.66). In response to the salt treatment, *TUB* and *TUA* (M = 0.71) had the most stable expression levels, whereas *18S* (M = 1.75) had the most unstable expression level. For the ABA treatment, *EF-1α* and *TUB* (M = 0.56) were detected as the most stably expressed genes and *GAPDH* (M = 1.26) was identified as the most variably expressed gene. Following the drought treatment, the expression levels were most stable for *ACT* and *18S* (M = 0.52) and least stable for *GAPDH* (M = 1.37). Overall, *EF-1α* and *TUA* (M = 0.73) were the most stably expressed genes in all samples, whereas *GAPDH* (M = 1.64) was the least stably expressed gene (Fig. 5).

**Figure 6:**
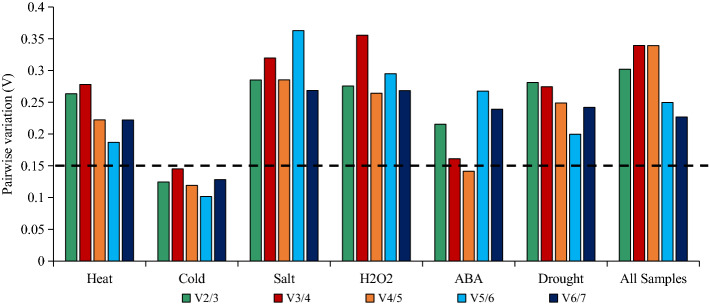
geNorm analysis of the V values for the seven reference genes under various conditions.

For optimal data normalization, two or more reference genes are necessary for qRT-PCR analyses. The optimal number of reference genes can be determined with the geNorm algorithm, which calculates the pairwise variation V_n_/V_n+1_. If V_n_/V_n+1_ is less than 0.15, n is the most suitable number of internal reference genes. However, if V_n_/V_n+1_ is greater than 0.15, n + 1 is the ideal number of internal reference genes. For the cold treatment, V_2_/V_3_ was less than 0.15 (Fig. 6), implying that two reference genes are sufficient for normalizing gene expression data. Regarding the ABA treatment, V_4_/V_5_ was less than 0.15, indicating that four reference genes should be used for the data normalization. For the other four treatments, V_n_/V_n+1_ exceeded 0.15, and the optimal number of reference genes was not determined.

### NormFinder analysis

NormFinder calculates the SV for each reference gene based on the intragroup and intergroup variations. Lower SVs correspond to higher gene expression stability. For the heat and ABA treatments as well as overall (i.e., all treatments), *EF-1α* and *GAPDH* were the most and least stably expressed reference genes, respectively. For the cold treatment, *EF-1α* was the most stably expressed reference gene, whereas *TUA* was the least stably expressed reference gene. Regarding the H_2_O_2_ and drought treatments, *TUB* and *GAPDH* were the most and least stably expressed reference genes, respectively. In response to the salt treatment, the most and least stably expressed genes were *UBQ* and *18S*, respectively (Table 2).

**Table 2:** Expression stability of seven reference genes following various treatments based on a NormFinder analysis.

Rank	Heat		Cold		Salt		H_2_O_2_	
	Gene	SV	Gene	SV	Gene	SV	Gene	SV
1	*EF-1α*	0.595	*EF-1α*	0.243	*UBQ*	0.454	*TUB*	0.470
2	*18S*	0.716	*TUB*	0.418	*TUA*	0.822	*EF-1α*	0.801
3	*ACT*	0.858	*18S*	0.420	*EF-1α*	1.034	*UBQ*	0.906
4	*TUA*	0.860	*GAPDH*	0.451	*TUB*	1.365	*ACT*	0.909
5	*UBQ*	0.892	*ACT*	0.522	*GAPDH*	1.482	*18S*	1.542
6	*TUB*	1.127	*UBQ*	0.698	*ACT*	1.745	*TUA*	1.728
7	*GAPDH*	1.508	*TUA*	0.876	*18S*	1.811	*GAPDH*	1.862

Rank	ABA		Drought		All samples	
	Gene	SV	Gene	SV	Gene	SV
1	*EF-1α*	0.282	*TUB*	0.438	*EF-1α*	0.829
2	*TUA*	0.289	*18S*	0.726	*TUB*	0.877
3	*TUB*	0.500	*UBQ*	0.743	*TUA*	1.055
4	*ACT*	0.726	*ACT*	1.000	*UBQ*	1.121
5	*18S*	1.019	*TUA*	1.076	*ACT*	1.331
6	*UBQ*	1.451	*EF-1α*	1.145	*18S*	1.432
7	*GAPDH*	1.640	*GAPDH*	1.654	*GAPDH*	1.519

### BestKeeper analysis

BestKeeper calculates the stability of candidate reference genes based on the SD, r, and CV of the Ct data for all reference genes. Low SD and CV values reflect stable gene expression. The rankings based on the BestKeeper analysis revealed that four reference genes were stably expressed following the heat treatment, of which *ACT* (CV ± SD = 0.59 ± 0.149) was the most stably expressed. For the cold treatment, all seven reference genes were stably expressed, but *ACT* (CV ± SD = 1.16 ± 0.298) and *EF-1α* (CV ± SD = 1.63 ± 0.342) were the most stably expressed. Regarding the salt treatment, the expression of four reference genes was stable, with *UBQ* (CV ± SD = 2.98 ± 0.56) identified as the most stably expressed gene. For the H_2_O_2_ treatment, four reference genes were suitable for normalizing gene expression data, among which *TUB* (CV ± SD = 2.13 ± 0.494) and *EF-1α* (CV ± SD = 2.7 ± 0.513) were the most stably expressed. In response to the ABA treatment, five reference genes were stably expressed, with *TUA* (CV ± SD = 1.9 ± 0.44) and *ACT* (CV ± SD = 1.87 ± 0.484) detected as the most stably expressed genes. Following the drought treatment, the expression levels of five reference genes were considerably stable, with *TUB* (CV ± SD = 2.25 ± 0.553) and *TUA* (CV ± SD = 2.46 ± 0.554) revealed as the most stably expressed genes. An analysis of all treatments indicated that four reference genes were stably expressed, with *ACT* (CV ± SD = 3.33 ± 0.875) identified as the gene with the most stable expression level (Table 3).

**Table 3:** Expression stability of seven reference genes following various treatments based on a BestKeeper analysis.

Rank	Heat			Cold			Salt			H_2_O_2_		
	Gene	SD	CV	Gene	SD	CV	Gene	SD	CV	Gene	SD	CV
1	*ACT*	0.149	0.59	*ACT*	0.298	1.16	*UBQ*	0.56	2.98	*TUB*	0.494	2.13
2	*TUA*	0.727	3.38	*EF-1α*	0.342	1.63	*18S*	0.883	4.31	*EF-1α*	0.513	2.7
3	*TUB*	0.746	3.08	*18S*	0.443	2.23	*ACT*	0.932	3.51	*ACT*	0.606	2.25
4	*EF-1α*	0.747	3.75	*GAPDH*	0.458	2.17	*TUA*	0.998	4.6	*UBQ*	0.777	3.99
5	*18S*	1.008	4.91	*TUA*	0.475	2.1	*EF-1α*	1.144	5.72	*TUA*	1.055	5.09
6	*UBQ*	1.325	6.66	*TUB*	0.495	2	*GAPDH*	1.288	6.23	*18S*	1.271	5.88
7	*GAPDH*	1.776	8.21	*UBQ*	0.657	3.24	*TUB*	1.534	6.71	*GAPDH*	1.288	6.39

Rank	ABA			Drought			All samples		
	Gene	SD	CV	Gene	SD	CV	Gene	SD	CV
1	*TUA*	0.44	1.9	*TUB*	0.553	2.25	*ACT*	0.875	3.33
2	*ACT*	0.484	1.87	*TUA*	0.554	2.46	*EF-1α*	0.957	4.71
3	*EF-1α*	0.617	2.9	*EF-1α*	0.794	3.84	*TUA*	0.958	4.34
4	*18S*	0.67	3.3	*ACT*	0.963	3.54	*TUB*	0.973	4.04
5	*TUB*	0.834	3.34	*18S*	0.966	4.63	*18S*	1.015	4.92
6	*GAPDH*	1.335	6.22	*UBQ*	1.062	5.25	*UBQ*	1.275	6.34
7	*UBQ*	1.604	7.27	*GAPDH*	1.34	5.87	*GAPDH*	1.443	6.77

### RefFinder analysis

Finally, RefFinder was used for the comprehensive ranking of the reference genes under each stress condition. The results indicated that *EF-1α* was the most stably expressed gene overall and for the heat, cold, and ABA treatments. The *UBQ* gene had the most stable expression level following the salt treatment, whereas *TUB* was the most stably expressed gene in response to the H_2_O_2_ and drought treatments. In contrast, *GAPDH* was the least stably expressed gene overall and for the heat, salt, H_2_O_2_, ABA, and drought treatments. Regarding the cold treatment, *TUA* was the least stably expressed gene (Table 4).

**Table 4:** Comprehensive ranking of stability.

Rank	Gene (Geomean of ranking values)						
	Heat	Cold	Salt	H_2_O_2_	ABA	Drought	All samples
1	*EF-1α*(1.41)	*EF-1α*(1.57)	*UBQ*(1.41)	*TUB*(1.41)	*EF-1α*(1.32)	*TUB*(1.41)	*EF-1α*(1.19)
2	*ACT*(2.28)	*TUB*(2.21)	*TUA*(2.00)	*UBQ*(2.45)	*TUA*(1.86)	*18S*(2.11)	*TUA*(2.28)
3	*TUA*(2.38)	*18S*(2.28)	*TUB*(3.25)	*EF-1α*(2.51)	*TUB*(2.59)	*ACT*(2.83)	*TUB*(2.63)
4	*18S*(3.16)	*ACT*(3.34)	*EF-1α*(3.41)	*ACT*(3.46)	*ACT*(3.36)	*UBQ*(3.57)	*ACT*(3.34)
5	*TUB*(4.56)	*GAPDH*(4.00)	*ACT*(5.05)	*18S*(3.50)	*18S*(4.73)	*TUA*(3.98)	*UBQ*(4.43)
6	*UBQ*(5.48)	*UBQ*(6.24)	*18S*(5.12)	*TUA*(5.73)	*UBQ*(6.24)	*EF-1α*(5.05)	*18S*(5.73)
7	*GAPDH*(7.00)	*TUA(*6.44)	*GAPDH*(5.23)	*GAPDH*(7.00)	*GAPDH*(6.74)	*GAPDH*(7.00)	*GAPDH*(7.00)

## Reference gene validation

**Figure 7:**
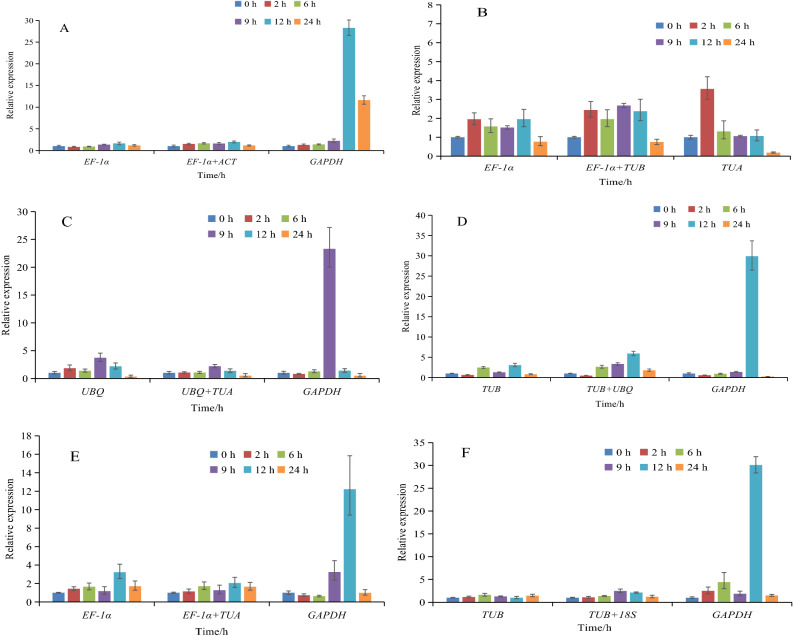
Expression of *LcCu/Zn-SOD1* gene under different stresses using validated reference genes for data normalization. (**A**) Expression of *LcCu/Zn-SOD1* gene under heat stress using validated reference genes *ACT*, *EF-1α*, and *GAPDH*. (**B**) Expression of *LcCu/Zn-SOD1* gene under cold stress using validated reference genes *TUA*, *EF-1α*, and *TUB*. (**C**) Expression of *LcCu/Zn-SOD1* gene under salt stress using validated reference genes *TUA*, *UBQ*, and *GAPDH*. (**D**) Expression of *LcCu/Zn-SOD1* gene under H_2_O_2_ stress using validated reference genes *TUB*, *UBQ*, and *GAPDH*. (**E**) Expression of *LcCu/Zn-SOD1* gene under ABA stress using validated reference genes *TUA*, *EF-1α*, and *GAPDH*. (**F**) Expression of *LcCu/Zn-SOD1* gene under drought stress using validated reference genes *TUB*, *18S*, and *GAPDH*.

To validate the accuracy of the analysis of reference gene expression stability, the relative expression levels of luffa *LcCu/Zn-SOD1* gene were analyzed under various stresses conditions. The two most stable reference genes and one unstable reference gene according to RefFinder were selected for normalizing gene expression data. The results indicated that when the most ideal reference genes were used alone or combined as the internal reference control, the expression of the *LcCu/Zn-SOD1* exhibited similar trends with minor changes. Moreover, the use of two reference genes generally improved the quantification of *LcCu/Zn-SOD1* gene expression. However, the *LcCu/Zn-SOD1* gene expression patterns were considerably different when the unstable reference gene was used to normalize data. Specifically, the expression levels were significantly higher at 9 or 12 h under heat, salt, H_2_O_2_, ABA and drought treatment conditions, reflecting the overestimation of gene expression levels at these two time-points (Fig. 7). Therefore, the use of inappropriate reference genes may lead to biased and incorrect expression analyses.

## Discussion

The qRT-PCR assay, which has been widely used for quantifying target gene transcription levels, is an important research tool applicable for characterizing gene functions^17,18^. However, the accuracy of gene expression analyses mainly depends on the selection of an appropriate internal control, which is often referred to as a reference or housekeeping gene. Reference genes were initially selected primarily based on housekeeping gene functions. For example, *ACT* and *TUB* genes encode the basic components of the cytoskeleton, whereas the proteins encoded by *GAPDH*, *EF-1α*, and *UBQ* genes contribute to the basic metabolic processes of organisms. Accordingly, these genes were expected to be stably expressed in all cells and physiological states^2^. Subsequent research proved that reference gene expression levels may vary in different tissues or growth and development stages. The expression might also be influenced by biotic and abiotic stresses and hormones. Thus, there are no internal reference genes that are stably expressed under all experimental conditions^19–22^. For example, *GAPDH* expression is highly stable in grape berries, but is relatively unstable in wheat^23,24^. Both *ACT* and *UBQ* are stably expressed in wheat, but not in tomato^24,25^. Gong et al.^26^ analyzed the expression stability of 18 candidate reference genes of Goji at different developmental stages and under drought stress condition. They observed that two novel reference genes *LbCML38* and *LbRH52* were more stably expressed than the commonly used reference genes. Liu et al.^27^ analyzed perennial ryegrass under high-temperature stress and identified *HIS3* and *eIF4A* as the most suitable reference genes. In another study, *EF-1α* and *UBQ* were detected as the most stable internal reference genes in potato and *Arabis alpina* under drought conditions^28,29^. Therefore, verifying the expression stability of reference genes under different experimental conditions is critical when selecting reference genes to standardize gene expression levels. However, there has been relatively little research regarding the systematic validation of reference genes in luffa. Only *18S* has been reported as a reference gene in luffa, but it has often been used without any proper verification, possibly resulting in inaccurate analyses of luffa gene expression patterns.

In this study, the expression stability of seven candidate reference genes (*ACT*, *TUA*, *TUB*, *EF-1α*, *GAPDH*, *UBQ*, and *18S*) after heat, cold, salt, H_2_O_2_, ABA, and drought treatments was analyzed with BestKeeper, geNorm, NormFinder, and RefFinder. The objective of this study was to identify the most stable reference genes for investigating gene expression in luffa plants exposed to abiotic stress factors. The data revealed *EF-1α* as the most stably expressed gene following the heat, cold, and ABA treatments. This gene, which encodes a polyribosomal protein, is widely expressed in various cells. In many studies on Cucurbitaceae species, *EF-1α* has been used as an appropriate reference gene. In cucumber, *EF-1α*, *Fbox*, *CAC*, and *TIP41* are reportedly stably expressed regardless of the abiotic stress, growth regulator treatment, and nitrogen status^30,31^. In another study on cucumber, *EF-1α* was confirmed as the most stably expressed reference gene^32^. In an earlier study on zucchini, *UFP*, *EF-1α*, *RPL36aA*, *PP2A*, and *CAC* were identified as the ideal reference genes for normalizing expression data^33^. Ye et al.^34^ screened and evaluated reference genes for fluorescence-based real-time quantitative PCR analyses of wax gourd and confirmed that *EF-1α* is the most stably expressed reference gene under cold conditions and in different tissues. In the current study, *UBQ* was the most stably expressed reference gene following the salt treatment, which is consistent with the results of an earlier study by Jin et al.^35^. The *UBQ* gene belongs to the ubiquitin gene family, which affects many important biological processes, including cell cycle regulation, growth and apoptosis, signal transduction, and immune responses. Because of its high sequence homology and the fact it is highly conserved, *UBQ* has been used as a reference gene for rice^36^, black fungus^37^, and cucumber^32^. Our data also indicated that *TUB* was the most stably expressed reference gene for the H_2_O_2_ and drought treatments. This gene is involved in plant growth and development, with an important role in maintaining cell morphology, promoting intracellular transport, and mediating cell movement and cell division, and has often been used as a reference gene. Kiarash et al.^38^ determined that *TUB* is the most appropriate reference gene for wheat plants under drought conditions. Although *ACT* is the most commonly used reference gene for cucurbit crops^39–41^, it was not identified as the best reference gene for any of the conditions analyzed in this study. Furthermore, *ACT* expression reportedly varies substantially in many other crops^42^. Kong et al.^20^ suggested that *ACT* should be used together with other reference genes for qRT-PCR analyses of melons. In the current study, except for the cold treatment, *GAPDH* was the most unstably expressed reference gene, implying that *GAPDH* is not a suitable reference gene for investigations of the effects of abiotic stresses on luffa gene expression. This is consistent with the results of studies by Liu et al.^27^ and Yu et al.^43^. Although *GAPDH* is the most unsuitable reference gene for annual ryegrass and ramie under different stress conditions, it is ideal for studies of *Juglans regia* L^44^, *L. speciosa*^45^ and *Stipa grandis*^46^. Therefore, potential internal reference genes must be evaluated and confirmed as appropriate for different experimental systems and materials before they are used to analyze gene expression.

To verify the reliability of the identified reference genes, we analyzed the expression of luffa *LcCu/Zn-SOD1* gene. Because of its protective effects on cell membranes, Cu/Zn-SOD can protect plants from oxidative damage due to reactive oxygen species, while also contributing to plant responses to various abiotic stresses^47^. In the present study, the expression of *LcCu/Zn-SOD1* gene initially increased in response to the abiotic stress, after which it decreased. This expression pattern was consistent with previously reported stress-induced expression trends^48–50^. Earlier studies tended to emphasize the use of a single reference gene for calibrating the expression of target genes. However, a single reference gene may be unstably expressed under different experimental conditions. Thus, applying multiple genes may increase the accuracy and reliability of data normalizations to some extent^51^. In this study, the combined use of two stably expressed reference genes generally enhanced the quantification of *Cu/Zn-SOD* gene expression when compared with the data obtained with a single reference gene. Deng et al.^22^ suggested that potential interactions between reference genes should be examined when using multiple reference genes. A positive or negative relationship between the expression of the reference genes may affect the analysis of the target gene expression level. Reference genes with linearly additive expression may be used together as internal reference controls to improve the normalization of expression data under various experimental conditions. Therefore, the method used to select multiple reference genes should be carefully considered to ensure appropriate combinations are applied.

## Methods

### Plant materials and treatments

‘Minyan No. 1’, a commercial F_1_ luffa hybrid, was used in the present study. The seeds were first wrapped with wet gauze and then germinated in an incubator at 30 °C. The seedlings were sown in 10 × 8 cm pots containing soil and cultivated in an artificial climate incubator with a 16-h artificial light (25 °C):8-h dark (18 °C) cycle and 65–75% relative humidity. Seedlings at the third true leaf stage were used for the following treatments. For the heat and cold treatments, the seedlings were incubated at 40 °C and 4 °C, respectively, for 24 h. For the ABA and H_2_O_2_ treatments, the seedlings were sprayed with 200 μM ABA and 100 μM H_2_O_2_, respectively. Regarding the salt and drought stress treatments, the seedlings were transplanted into full-strength Hoagland’s nutrient solution containing 100 mM NaCl or 15% PEG 6000, respectively, for 24 h. Leaves were then collected at 0, 2, 6, 9, 12, and 24 h after the treatments. Each experiment was completed with three replicates, each comprising five seedlings. All collected leaf samples were immediately frozen in liquid nitrogen and stored at − 80 °C until analyzed.

### RNA extraction and cDNA synthesis

Total RNA was extracted from the frozen leaf samples with the MiniBEST Plant RNA Extration Kit (TaKaRa, Dalian, China). The NanoDrop ND1000 spectrophotometer (Thermo Scientific, Wilmington, DE, USA) was used to calculate the RNA concentration and assess purity. The RNA samples with a 260/280 nm absorbance ratio of 1.8–2.0 were retained for further analyses. The RNA integrity was evaluated by 1% agarose gel electrophoresis. The PrimeScript II First Strand cDNA Synthesis Kit (TaKaRa) was used to synthesize cDNA.

### Reference gene isolation and sequence analysis

Transcripts for five potential reference genes (*ACT*, *TUA*, *TUB*, *EF-1α*, and *GAPDH*) were identified following a search of an established luffa transcriptome database. Complete open reading frames (ORFs) were obtained for all sequences. Gene-specific primers were designed to clone and verify the ORF sequences of the five genes (Table 5). The PCR amplification was completed in a reaction solution consisting of 1 μL cDNA (100 ng), 1 μL 0.4 μM forward primer, 1 μL 0.4 μM reverse primer, 0.5 μL 0.15 mM dNTPs, 0.2 μL 1 U Taq DNA polymerase, 2.5 μL 1.5 mM 10 × PCR buffer, and 18.8 μL ddH_2_O for a final volume of 25 μL. The PCR program was as follows: 94 °C for 3 min; 35 cycles of 94 °C for 30 s, 54 °C for 30 s, and 72 °C for 90 s; 72 °C for 7 min. The PCR products were analyzed by 1% agarose gel electrophoresis. They were then purified and inserted into the pMD-19T vector and sequenced. The homology between the five potential reference gene nucleotide sequences and known sequences was determined with the NCBI BLAST algorithm.

**Table 5:** Primers used for cloning the reference gene open reading frames.

Gene	Forward primer, 5′–3′	Reverse primer, 5′–3′
*TUB*	5′-ACCGTGAGAAGATGAGGGAA-3'	5′-GTTACTAATTGTCGAGGTCC-3'
*EF-1α*	5′-AGGCTGCTTATTTCTACGGA-3'	5′-AAATGCAGGCTTGGCTGGTT-3'
*GAPDH*	5′-CCCTGAATCCCCACTTCTTT-3'	5′-AAACTAACCCATGGTGTAGG-3'
*ACT*	5′-CTTCGAGCCAAATCGCTTTC-3'	5′-TCTCAGGTAAAACCGTACCG-3'
*TUA*	5′-TATTCTCAGAGGCACACTCG-3'	5′-AACGGCAGGCTCTTGAACTA-3'

### Reference gene selection and primer design

The seven genes initially selected as candidate reference genes included the *ACT*, *TUA*, *TUB*, *EF-1α*, and *GAPDH* genes as well as two previously cloned reference genes, *UBQ*^16^ and *18S*^15^. Details regarding these genes are provided in Table 1. All qRT-PCR primers were designed with the Primer 5.0 software. The specificity of each primer pair was evaluated by 1% agarose gel electrophoresis and a melting curve analysis. The PCR amplification efficiency (E) and correlation coefficient (R^2^) for each qRT-PCR primer pair were calculated with a fivefold cDNA dilution series^52^.

### qRT-PCR analysis

A qRT-PCR assay was completed with SYBR Premix Ex Taq (TaKaRa) and the QuantStudio 7 Flex Real-Time PCR System (Applied Biosystems, USA). The reaction solution consisted of 10 μL SYBR Green PCR Master Mix (TaKaRa), 2 μL cDNA (100 ng), 0.8 μL 10 µM forward primer, 0.8 μL 10 µM reverse primer, 0.4 μL ROX Reference Dye II, and 6 μL ddH_2_O for a final volume of 20 μL. The amplification conditions were as follows: 95 °C for 30 s; 40 cycles of 95 °C for 5 s, 60 °C for 34 s, and 72 °C for 10 s, followed by a melting curve analysis from 65 to 95 °C. A blank control lacking a template was also analyzed. The gene expression levels for each sample were determined based on three replicates.

### Data analysis

The cycle threshold (Ct) value was recorded for each qRT-PCR analysis of gene expression. Box plots were drawn to visualize the reference gene expression levels and variations. The geNorm, NormFinder, BestKeeper, and RefFinder algorithms were used to assess the expression stability of selected genes. Specifically, geNorm calculates the expression stability value (M) and pairwise variation (V), and the most stably expressed gene is the one with the lowest M value. Moreover, geNorm determines the optimal number of reference genes according to the relative value V_n_/V_n+1_. NormFinder calculates a stability value (SV) for each gene based on the variance analysis, and the gene with the lowest SV is identified as the most stably expressed gene. For both algorithms, the Ct values should first be transformed by 2^−ΔCt^. BestKeeper mainly determines the stability of reference gene expression based on the standard deviation (SD), Pearson correlation coefficient (r), and the coefficient of variation (CV) of the Ct data for all reference genes. The most stably expressed gene is the one with the lowest SD and CV values. Finally, the reference genes were ranked based on the geometric mean (GM) values calculated with RefFinder (http://www.leonxie.com/referencegene.php).

### Validation of reference genes

To confirm the reliability of the reference genes, the expression profiles of *Cu/Zn-SOD* gene were determined and normalized against the data for the two most stable and the least stable reference genes^22^. The qRT-PCR amplification conditions were as described above and the primer pairs are listed in Table 1. The relative expression data were calculated with the 2^−ΔΔCt^ method^53^. Statistical analyses were performed with the SPSS 18 program.

### Ethics approval and consent to participate

Not applicable.

### Consent for publication

All authors have consented for publication.

## Supplementary Information

Supplementary Information

## Data Availability

The datasets supporting the conclusions and description of a complete protocol are included within the article.
