# Supplementary material for: Reference gene selection for qRT-PCR analyses of luffa (Luffa cylindrica) plants under abiotic stress conditions
Source: Sci Rep. 2021 Feb 4;11:3161. doi: 10.1038/s41598-021-81524-w (PMC7862638; doi:10.1038/s41598-021-81524-w)
Supplement: Supplementary file 1 — Supplementary Information [file 41598_2021_81524_MOESM1_ESM.docx]

**Reference gene selection for qRT-PCR analyses of luffa (*Luffa cylindrica*) plants under abiotic stress conditions**

Min-dong Chen, Bin Wang, Yong-ping Li, Mei-juan Zeng, Jian-ting Liu, Xin-ru Ye, Hai-sheng Zhu & Qing-fang Wen

Fujian Key Laboratory of Vegetable Genetics and Breeding, Crops Research Institute, Fujian Academy of Agricultural Sciences, Vegetable Research Center, Fujian Engineering Research Center for Vegetables, Fuzhou, Fujian 350013, China. Corresponding author email: zhs0246@163.com.


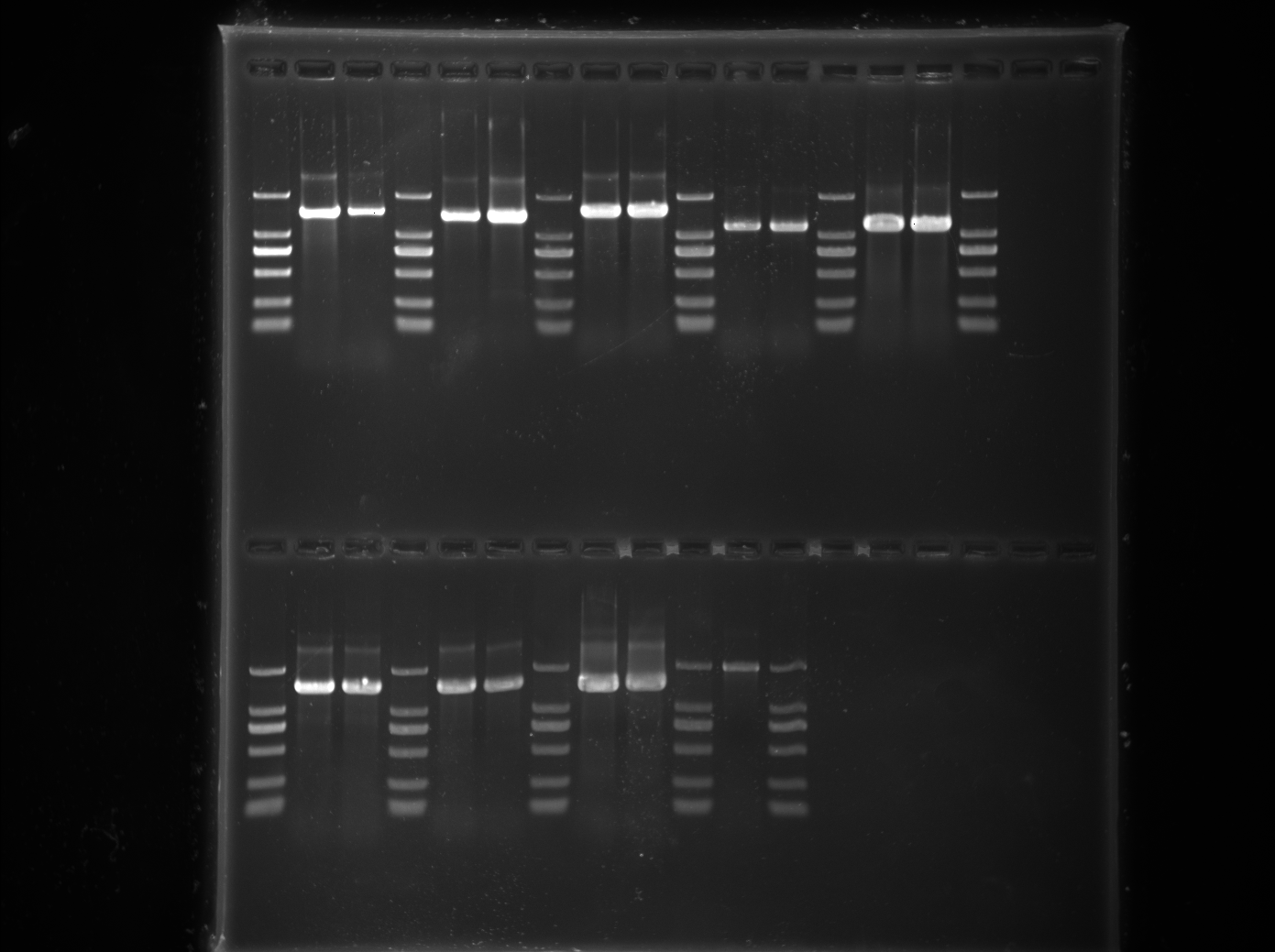


M A

**Supplementary Fig.1** The full-length agarose gel electrophoresis of Fig.1(A)


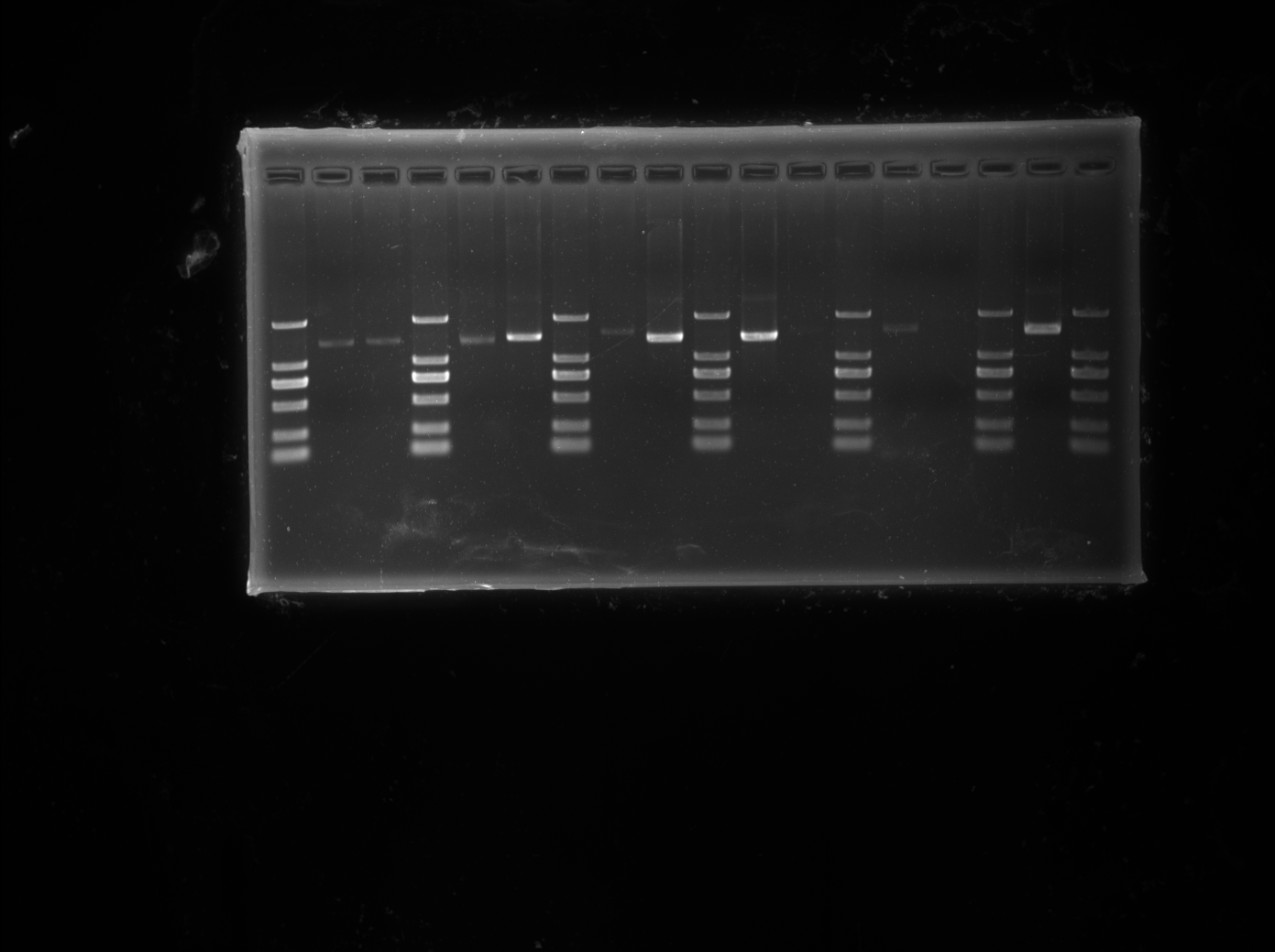


B M E D M C

**Supplementary Fig.2** The full-length agarose gel electrophoresis of Fig.1(B-E)

| Reference genes  Treatments | | Ct value | | | | | | |
| --- | --- | --- | --- | --- | --- | --- | --- | --- |
|  |  | *UBQ* | *TUB* | *EF-1α* | *GAPDH* | *ACT* | *TUA* | *18S* |
| Heat | 0 h | 19.034 | 23.745 | 20.536 | 20.714 | 25.220 | 22.640 | 19.613 |
|  | 2 h | 17.160 | 23.648 | 18.567 | 19.367 | 24.991 | 20.918 | 19.061 |
|  | 6 h | 19.514 | 24.254 | 18.980 | 19.761 | 25.351 | 20.298 | 20.864 |
|  | 9 h | 20.780 | 24.823 | 20.463 | 21.365 | 25.666 | 21.240 | 19.931 |
|  | 12 h | 21.517 | 25.933 | 20.072 | 24.373 | 25.320 | 22.198 | 21.640 |
|  | 24 h | 21.364 | 23.148 | 20.743 | 24.224 | 25.409 | 21.983 | 22.148 |
| Cold | 0 h | 19.034 | 23.745 | 20.536 | 20.714 | 25.220 | 22.640 | 19.613 |
|  | 2 h | 19.627 | 24.801 | 20.950 | 21.726 | 25.673 | 23.919 | 19.742 |
|  | 6 h | 20.267 | 24.774 | 20.935 | 21.118 | 25.730 | 22.773 | 19.931 |
|  | 9 h | 21.275 | 25.941 | 21.085 | 20.773 | 25.568 | 22.677 | 20.864 |
|  | 12 h | 20.358 | 24.362 | 20.597 | 20.368 | 26.262 | 21.818 | 18.936 |
|  | 24 h | 21.239 | 25.108 | 21.964 | 21.761 | 26.176 | 22.059 | 20.155 |
| Salt | 0 h | 19.034 | 23.745 | 20.536 | 20.714 | 25.220 | 22.640 | 19.613 |
|  | 2 h | 17.921 | 20.275 | 17.628 | 18.406 | 27.687 | 19.920 | 22.312 |
|  | 6 h | 17.967 | 21.381 | 20.501 | 19.524 | 27.975 | 20.816 | 21.298 |
|  | 9 h | 19.613 | 23.885 | 20.084 | 23.941 | 26.716 | 21.697 | 20.308 |
|  | 12 h | 19.177 | 22.370 | 18.945 | 20.219 | 25.588 | 21.441 | 19.196 |
|  | 24 h | 18.992 | 25.600 | 22.324 | 21.221 | 25.976 | 23.789 | 20.156 |
| H_2_O_2_ | 0 h | 19.034 | 23.745 | 20.536 | 20.714 | 25.220 | 22.640 | 19.613 |
|  | 2 h | 17.667 | 23.233 | 18.724 | 20.073 | 26.753 | 21.959 | 19.830 |
|  | 6 h | 19.463 | 23.966 | 18.895 | 19.538 | 26.888 | 20.564 | 22.526 |
|  | 9 h | 20.712 | 22.631 | 18.810 | 19.721 | 27.560 | 20.183 | 22.609 |
|  | 12 h | 20.397 | 23.190 | 18.535 | 23.460 | 27.742 | 19.058 | 22.731 |
|  | 24 h | 19.720 | 22.213 | 18.485 | 17.425 | 27.198 | 19.902 | 22.459 |
| ABA | 0 h | 19.034 | 23.745 | 20.536 | 20.714 | 25.220 | 22.640 | 19.613 |
|  | 2 h | 20.521 | 24.578 | 20.792 | 20.007 | 25.832 | 22.255 | 21.062 |
|  | 6 h | 21.833 | 25.068 | 20.880 | 19.686 | 26.092 | 23.091 | 20.372 |
|  | 9 h | 23.709 | 24.016 | 21.068 | 22.574 | 25.669 | 23.397 | 19.558 |
|  | 12 h | 23.121 | 25.795 | 22.333 | 24.365 | 25.223 | 23.129 | 19.716 |
|  | 24 h | 24.183 | 26.482 | 22.080 | 21.479 | 27.056 | 24.099 | 21.476 |
| Drought | 0 h | 19.034 | 23.745 | 20.536 | 20.714 | 25.220 | 22.640 | 19.613 |
|  | 2 h | 18.772 | 24.337 | 20.285 | 22.422 | 26.551 | 22.255 | 20.002 |
|  | 6 h | 19.704 | 24.710 | 20.745 | 23.124 | 26.819 | 23.091 | 20.106 |
|  | 9 h | 20.976 | 23.841 | 18.767 | 21.354 | 28.529 | 21.617 | 21.609 |
|  | 12 h | 21.005 | 24.689 | 20.896 | 26.561 | 28.527 | 21.971 | 22.726 |
|  | 24 h | 21.904 | 25.840 | 22.709 | 22.847 | 27.310 | 23.433 | 21.184 |

Supplementary Table S1 The Ct values for all samples in this study

Note: The Ct values for cold, drought, heat, salt, H_2_O_2_, ABA treatments in this study. Low Ct values reflect high expression levels.
